# A Study of Financial Incentives to Reduce Plasma HIV RNA Among Patients in Care

**DOI:** 10.1007/s10461-013-0416-1

**Published:** 2013-02-13

**Authors:** Steven Farber, Janet Tate, Cyndi Frank, David Ardito, Michael Kozal, Amy C. Justice, R. Scott Braithwaite

**Affiliations:** 1 Department of Medicine, Connecticut VA Healthcare System, West Haven, CT USA; 2 Department of Medicine, Yale University, New Haven, CT USA; 3 Division of Comparative Effectiveness and Decision Science, New York University Langone Medical Center, 227 East 30th Street, TRB, 6th Floor, New York, NY 10016 USA

**Keywords:** Financial incentives, Adherence, Antiretroviral therapy, HIV/AIDS

## Abstract

The role of financial incentives in HIV care is not well studied. We conducted a single-site study of monetary incentives for viral load suppression, using each patient as his own control. The incentive size ($100/quarter) was designed to be cost-neutral, offsetting estimated downstream costs averted through reduced HIV transmission. Feasibility outcomes were clinic workflow, patient acceptability, and patient comprehension. Although the study was not powered for effectiveness, we also analyzed viral load suppression. Of 80 eligible patients, 77 consented, and 69 had 12 month follow-up. Feasibility outcomes showed minimal impact on patient workflow, near-unanimous patient acceptability, and satisfactory patient comprehension. Among individuals with detectable viral loads pre-intervention, the proportion of undetectable viral load tests increased from 57 to 69 % before versus after the intervention. It is feasible to use financial incentives to reward ART adherence, and to specify the incentive by requiring cost-neutrality and targeting biological outcomes.

## Introduction

Financial incentives may influence behaviors that are resistant to change by providing positive reinforcement [1]. Effective financial incentives employ basic principles of behavior reinforcement, including identification of a target behavior, frequent collection of an objective measure of that behavior, selection of desirable reinforcement, and consistent and immediate link between target behavior and reinforcers [1]. While financial incentives have been most commonly studied to reduce substance misuse [2–9], they are increasingly evaluated in other areas where therapeutically relevant behaviors may be targeted, including adherence with prescribed medications [10, 11]. However, using financial incentives to increase adherence to antiretroviral therapy (ART) is not well studied, even though finding new interventions to improve ART adherence is of paramount importance to public health. ART nonadherence remains highly prevalent [12] despite the reduced pill burden and side effects of newer regimens. ART nonadherence is an important cause of preventable morbidity and mortality among HIV-infected patients, and is a major cause of HIV transmission [13, 14].

Accordingly, we sought to conduct a pilot study to evaluate the feasibility of using a financial incentive to improve ART adherence. Our study was motivated by several design goals that are unusual in studies of financial incentives [2–9]: First, to address concerns that financial incentives are not sustainable or scalable because they are too expensive [1], the size of the incentive was designed to be cost-neutral or cost-saving, guided by a calculation regarding downstream HIV costs averted by preventing HIV transmission through viral load reduction. Second, to link the incentive tightly to preventable morbidity and mortality, the primary target of the incentive was viral load suppression rather than a more direct measure of adherence. Third, to be easily understood and accepted by the target population (e.g., patients of lower education and socioeconomic status), the incentive algorithm rewarded improvement as well as achievement, was not easily game-able, yet was concise and transparent.

## Methods

### Intervention

Our study intervention consisted of a monetary payment ($100) contingent on an “either/or” reward criterion: patients needed to either (1) suppress their plasma HIV RNA below the lower limit of detection of the assay used in our clinic (Roche HIV—1 Monitor Cobas, 48 copies/ml), or (2) demonstrate a viral load that is at least one log10 lower than their prior lowest viral load in the past year (Table 1).

**Table 1:** Calculation of health costs averted by reducing probability of HIV transmissions

Pre-ART transmission rate per person year	Assuming no viral load suppression from partial ART adherence			Assuming substantial viral load suppression from partial ART adherence		
	Reduction in transmissions per person year by improving adherence	Cost saved per year	Cost saved per quarter	Reduction in transmissions per person year by improving adherence	Cost saved per year	Cost saved per quarter
0.01	0.00592	$2,134	$533	0.00124	$448	$112
0.02	0.01184	$4,268	$1,067	0.00249	$896	$224
0.05	0.02960	$10,670	$2,668	0.00622	$2,241	$560
0.10	0.05920	$21,341	$5,335	0.01240	$4,482	$1,120
0.20	0.11840	$42,683	$10,671	0.02490	$8,963	$2,241

We calculated the reduction in annual probability of transmitting HIV for a person who knows his serostatus and has typical risk behavior, and multiplied this estimation by the downstream HIV costs avoided by averting a new HIV infection ($360,500, based on an inflation-updated version of the estimate by Schackman et al. [15]). We performed calculations alternatively assuming (1) no viral load suppression from partial ART adherence below an assumed pre-ART baseline of 4.4 log units, and (2) substantial viral load suppression from partial adherence to a level 1 log unit above the assay detection threshold. We performed calculations for pre-ART transmission rates across a wide range of risk behavior profiles informed by recent United States estimates (0.01 transmissions per person per year, lowest, to 0.20 transmissions per person per year, highest) [23]. Our base case assumption (0.01 transmissions per person per year) was very conservative, below the transmission rate observed in HPTN 052 (0.017 transmissions per person per year) [14] or most recent estimates of the United States HIV transmission rate (0.041 transmissions per person per year) [24]. We assumed that each log 10 decrease in viral load below 4.4 decreased infectivity by 59 %, based on results from the Rakai study [25], and consistent with more recent results from HPTN 052 (65 %) [14]. Our most conservative estimate for costs averted per quarter ($112) was used as the basis of our incentive payment ($100)

#### Size of Incentive

We chose $100 as the size of the incentive because it was a round figure that approximated the minimum future health costs averted by reducing viral load by one log10 unit ($112, Table 1), based on assuming an extremely conservative (that is, lower bound) pre-ART transmission rate of 0.01 per person per year. We based our calculation on an extremely conservative transmission risk to pre-empt any concern that the incentive was not cost-neutral or cost-saving. We estimated health costs averted by calculating the reduction in annual probability of transmitting HIV for a person who knows his/her serostatus, has typical risk behavior, has substantial viral load suppression due to partial adherence and with an additional potential decrease of 1 log unit with perfect adherence (Table 1). We then multiplied this estimation by the downstream HIV costs avoided by averting a new HIV infection ($360,500 in 2012 US dollars) [15]. Therefore, the financial incentive, while substantial, and in line with other incentives that impact behavior resistant to change (e.g., smoking) [16], would not increase health care costs over long time horizons, an attribute that may enhance its scalability and sustainability.

#### Target of Incentive

We chose to make the target of the incentive a clinical outcome (viral load suppression) rather than the behavior itself (ART adherence) because we sought a target that was closely linked to preventable morbidity and mortality, and because viral load is a more objective measure than most measures of adherence (e.g., for example, medication bottles with sensors can be received and opened, but medication may not be taken). Additionally, it can be argued that the vast majority of variability in viral load is attributable to variations in patient adherence, and much of the variability that is not attributable to patient adherence is attributable to ineffective ART regimens, which can also typically be affected through patient behavior (e.g., working with their clinicians to find a more effective ART regimen). For these reasons, we thought it was reasonable to link the incentive to the clinical outcome rather than to the behavior itself. However, we noted that this approach may not be suited to individuals who have multidrug resistance.

#### Criteria for Incentive

We chose an “either/or” reward criterion (undetectable viral load OR demonstration of a viral load that is at least one log10 lower than their prior lowest viral load in the past year; Table 2) because we wanted to reward improvement as well as achievement. We chose the last year as a comparator, rather than a single test result or a shorter time interval, to avoid the possibility that patients would “game” the incentive by deliberately alternating periods of poorer adherence with periods of better adherence. To avoid the possibility of patients seeking multiple payments for the same quarter, a patient could not qualify for more than one incentive payment in a 3 month window, an interval which corresponds to the usual frequency of viral load measurement in the clinic. Patients were not allowed “second chances” to earn the incentives during a 3 month window, and therefore blood tests were not repeated at the patient’s request until the next quarter, unless desired by the patient’s clinician. Thus the study required no additional blood tests.

### Inclusion and Exclusion Criteria

We targeted the financial incentive towards individuals with detectable viral load during the last year, and used each patient as his own control. Consequently, our initial inclusion criteria were any individual enrolled in clinic who was receiving ART for at least 1 year, had at least one detectable viral load within the last year, and who was capable of giving informed consent. We did not supplement the historical controls with an additional concurrent control group because this was not an effectiveness study, and because there were insufficient numbers of patients to make this a feasible option. We broadened our initial inclusion criteria when concerns were raised by the clinic director and the IRB concerning the fairness of the study. In particular, if only patients with detectable viral load were eligible for study inclusion, and therefore only patients with detectable viral load were eligible for receiving the financial incentives, this criterion could be viewed as penalizing people who had achieved satisfactory viral load control during the past year. Based on this concern, we broadened study inclusion criteria to include all patients receiving ART in the past year regardless of viral load detectability; however, we pre-specified the subgroup of patients with at least one detectable viral load as the target population of greatest interest.

Each participant was briefed about whether they did or did not qualify for the incentive by the study research coordinator, not by the patient’s clinician. The study coordinator initiated a scripted discussion, using the incentive schedule (Table 2) as a visual prompt, and reinforcing the assessment when necessary by reviewing the patient’s lab values over the past year. These visits could either occur before or after the patients’ scheduled clinician visit. Most study visits occurred immediately before or after the clinician visit, although patients were permitted to receive their briefing on a later day if they wanted to leave the clinic immediately. While the patient’s clinician was allowed to discuss laboratory results as part of normal care during the visit, she was discouraged from revealing or discussing whether or not the patient qualified for the incentive.

**Table 2:** Algorithm to determine whether patients qualified for incentive payments

Viral load	Grade
Undetectable	A
50–499	B
500–4,999	C
5,000 or above	D

If your best grade in the last year was	Grade needed for incentive
D	C
C	B
B	A
A	A

### Outcomes

The intervention was applied for 1 year (4 quarters). Feasibility outcomes were (1) impact on clinic workflow, (2) patient acceptability, and (3) patient comprehension. We assessed impact on clinic workflow by interviewing clinicians who had patients that participated in the study. We assessed patient acceptability by recording instances when patients were unsatisfied or disappointed as a consequence of the study intervention (e.g., they did not receive the incentive, yet they thought they would or should). We assessed patient comprehension by asking the study coordinator about whether patients understood the incentive system, as well as whether they could correctly predict whether they qualified for it based on their most recent lab result.

Although the study was not powered for effectiveness, we performed exploratory analyses of viral load suppression and ART adherence using each patient as his own control, comparing the intervention year with the prior year. We compared (1) proportion of viral load measurements that were undetectable, (2) area under the curve (AUC) viral load during the last year, and (3) ART adherence, assessed by number of ART medication fills during the period of analysis.

**Fig. 1:**
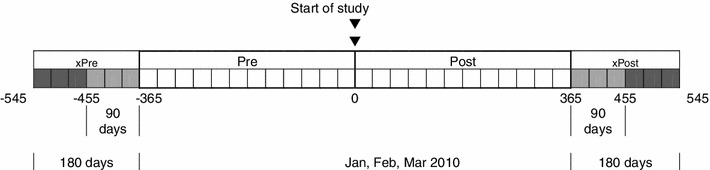
Time course of study

HIV-1 RNA tests done during the two years were used to assess the proportion of tests with undetectable virus. The limit of detection during the entire study was 48 copies/ml. Because results were not always available for the exact start and end dates, additional viral load tests 90 days beyond the start and end of the two year period (Fig. 1) were obtained. These were used to estimate the level of virus at the start of the prior year, at the start of the intervention, and at the end of the intervention. Estimates were made by interpolating values obtained on dates on either side of the target date. These were then used in AUC analysis.

AUC viral load is a measure of a person’s cumulative exposure to HIV-1 RNA over time [17]. The AUC is determined using the trapezoid rule to approximate the area under each patient’s longitudinal curve of HIV-RNA versus time. For each segment, the mean of the two measurements is multiplied by the number of days between measurements to determine area. Individual areas are summed to create the AUC. To account for differences in the number of days included the area is divided by the number of days giving AUC/day. Undetectable viral loads were assumed to have a value of 48 copies/ml.

### Statistical Methods

All eligible patients were included in “intent-to-treat” analysis. Only those that completed the entire intervention year were included in “as-treated” analysis. Proportions were compared using the χ^2^ test; means were compared with the paired sample *t* test. All analyses were performed with SAS version 9.2 (SAS institute, Cary, NC). No imputation was performed for patients who were lost-to-follow up or who died in the intent-to-treat analysis.

## Results

Of 80 eligible patients, 77 consented to participate in the incentive program (Table 3), and 69 were available for 12 month follow-up (3 died; 3 relocations; 2 lost to follow-up). Median age was 59, all were male, 62 % were nonwhite, and 48 % had history of alcohol dependence (assessed by inclusion in the problem list of the electronic health record and/or presence of corresponding ICD-9 diagnostic codes), 52 % had history of injection drug use, and 34 % had history of depression. 52 % (*N* = 40) had ≥1 detectable viral load during the year prior to the incentive. Most patients (59 %) were on PI-based regimens, with a minority on NNRTI-based regimens (29 %) or other regimens (12 %). Patients were treatment experienced, with 60 % on third or later ART regimens. Of the 51 patients (74 %) with genotypic resistance testing, 33 (65 %) had wild-type virus, 9 (18 %) had one-class resistance, and 9 (18 %) had two or more-class resistance. All but 5 patients were on ART 2 years prior to enrollment and all but 1 patient was on ART 18 months prior to enrollment.

**Table 3:** Characteristics of patients enrolled in study

	All enrolled		Completed		*p*	Test statistic	Test value	Detail
	*N* = 77		*N* = 69					
	*N*	(%)	*N*	(%)				
Enrolled					0.24	χ^2^	2.9	χ^2^
January	37	48.1	33	47.8				
February	24	31.2	20	29				
March	16	20.8	16	23.2				
Race					0.62	χ^2^	1.0	χ^2^
Black	41	53.2	36	52.2				
White	29	37.7	26	37.7				
Unknown	7	9.1	7	10.1				
Ethnicity					0.78	χ^2^	0.5	χ^2^
Non-hispanic	69	89.6	62	89.9				
Hispanic	6	7.8	5	7.2				
Other	2	2.6	2	2.9				
Detectable virus in year prior to enrollment	40	51.9	33	47.8	0.03	χ^2^	4.5	χ^2^
Log HIV RNA at enrollment, mean (SD)	1.9	(0.56)	1.8	(0.31)	0.24	*t*	1.3	*t* test, Satterthwaite for unequal variances
CD4 at enrollment, mean (SD)	503	(240)	517	(236)	0.12	*t*	−1.6	*t* test, pooled variance
Year of HIV diagnosis					0.83	χ^2^	0.4	χ^2^
<1990	15	19.5	13	18.8				
1990–1999	36	46.8	32	46.4				
2000+	26	33.8	24	34.8				
Median (IQR)	1996	(1992–2002)	1996	(1992–2002)				
Years since diagnosis					0.66	χ^2^	0.8	χ^2^
<10 years	20	26	19	27.5				
10–19 years	41	53.2	36	52.2				
20+ years	16	20.8	14	20.3				
Median (IQR)	16	(9–19)	15	(9–19)				
Antiretroviral therapy regimen					0.44	χ^2^	1.6	χ^2^
First	11	(14.3)	11	(15.9)				
Second	20	(26.0)	18	(26.1)				
Third or more	46	(59.7)	40	(58.0)				
Injection drug use					0.60	χ^2^	1.0	χ^2^
None	30	39	27	39.1				
History	40	51.9	35	50.7				
Unknown	7	9.1	7	10.1				
Alcohol dependence					0.65	χ^2^	1.6	χ^2^
None	29	37.7	25	36.2				
History	37	48.1	33	47.8				
Current	4	5.2	4	5.8				
Unknown	7	9.1	7	10.1				
Hepatitis C infected	41	53.2	35	50.7	0.19	χ^2^	1.7	χ^2^

### Feasibility Outcomes

The intervention did not seem to have an adverse effect on clinic workflow. Patients were able to learn whether they qualified for the incentive either before or after their clinician visit. Time required for the qualification updates was approximately 5–10 min. No clinician expressed frustration about the impact of the study on workflow as the research activity occurred after and separate from the patient visit. The clinic administrator was moderately concerned at the beginning of the study about workflow challenges, but by the end of the study, she no longer had concerns.

The incentive was acceptable to all patients except for one, who expressed frustration after his first quarter of participation because he did not qualify for the incentive. His frustration was exhibited verbally, but without any threatening words or actions. However, he chose to continue participating in the study, and during the subsequent quarter he qualified for the incentive, and became pleased with the intervention. Occasionally patients had to wait to be seen by study personnel after their clinician visit, but this was generally not an issue of concern.

Patients generally appeared to understand the incentive system, and could correctly predict whether or not they qualified for it based on their current and prior laboratory results. There were no complaints about unfairness or lack of transparency. However, during the first quarter, there were some confusion about the timing of, and eligibility for the incentives. Some patients who did not qualify for incentive expressed concern that although they were adherent with all antiretroviral therapy, clinic appointments, and study requirements, they were disappointed that they were not able to receive at least a portion of the incentive (i.e. those with improved but still low detectable HIV viral load). These concerns were generally resolved by the second quarter through education by the research staff.

No patients expressed concerns that the incentive targeted a clinical outcome rather than a behavior itself, and there was acceptance of the premise that they could control the viral load by taking their medications with greater regularity, and/or working with their clinician to find a more effective drug regimen.

### Effectiveness Outcomes

**Fig. 2:**
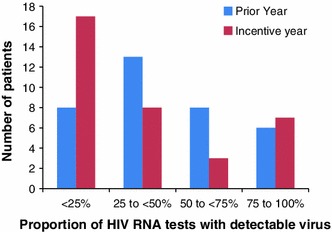
Number of patients stratified by proportion of HIV RNA tests with detectable viral load in the incentive year and in the year prior to the incentive (Color figure online)

Among the patient subgroup targeted by the study (individuals with prior detectable viral loads), the proportion of undetectable viral load tests increased from 57 to 69 % before versus after the intervention (χ^2^ = 4.7, *p* = 0.03) for intent-to-treat analyses, and 59 to 71 % in the as-treated patients (χ^2^ = 4.1, *p* = 0.04). Patients without any undetectable viral loads did not appear to be impacted by the intervention, whereas patients with occasionally undetectable viral loads appeared to have greater proportions of undetectable viral load tests after the intervention (Fig. 2). There was no evidence of “adherence fatigue”, with the proportion of undetectable viral loads rising throughout the intervention period (64 %, first quarter; 66 %, second quarter; 69 %, third quarter; and 78 %, fourth quarter).

AUC/day viral load decreased from 2.2 (95 % confidence interval, 1.7–2.6) to 1.9 (1.8–2.0) for intent-to-treat (*t* = −1.3, *p* = 0.19) and from 2.1 (1.7–2.6) to 1.9 (1.8–1.9) in the as-treated (*t* = −1.2, *p* = 0.23), but did not reach statistical significance with the number of patients available. ART adherence increased from a mean of 18.8 fills to 20.4 (*t* = 2.3, *p* = 0.02) before versus after the intervention for intent-to-treat, and 19.3–21.0 (*t* = 2.4, *p* = 0.02) as-treated. The correlation between increase in fills and decrease in AUC/day viral load was *r* = −0.16 (*p* = 0.21) in intent-to-treat analysis and was *r* = −0.21 (*p* = 0.10) in as-treated patients.

Among all patients enrolled in the study, there was no evident change in proportion of undetectable viral loads (76 vs. 77 %, before versus after the intervention) or AUC viral load, (2.0 versus 1.9, before versus after the intervention). Among the subgroup of patients who had no detectable viral loads prior to the study period (e.g., those who inclusion criteria were expanded to because of fairness concerns), 85 % of viral loads were undetectable during the study period. There was no evidence that patients on different ART regimens were differentially impacted by the intervention. Among patients on PI-based regimens, 50 % had undetectable viral loads after the intervention, whereas among patients on NNRTI-based regimens, 48 % had undetectable viral loads after the intervention.

## Discussion

This single-site study demonstrates the feasibility of implementing financial incentives aimed at reducing viral load in HIV patients in care. While other studies have investigated financial incentives in substance-using populations, this is the first study of contingency management in an HIV population that is not selected on the basis of active or prior substance abuse. Our study demonstrates the feasibility of designing and implementing a financial incentive system in which the size of the incentive was designed to be cost-neutral or cost-saving, the primary target of the incentive was the clinical outcome of interest (e.g., viral load suppression) rather than the behavior itself (e.g., adherence to ART), and the incentive algorithm rewarded improvement as well as achievement yet was difficult to “game.”

While our study was not powered for effectiveness outcomes, it raises the possibility that the incentive payments contributed to a 12 % increase in undetectable viral loads (from 57 to 69 %) among the target population. However, there are many other possible explanations for the finding, especially given the limitations of this small, single-site study, such as regression-to-the-mean. Follow-up studies can use more robust experimental designs to assess effectiveness. Additional questions that may be addressed by follow-up studies include whether the incentive can be implemented in different settings, and whether the incentive could be implemented using different amounts, schedules, and levels of certainty (e.g., lottery versus certain payment) [18–22]. Additional important limitations of the study include the large prevalence of patients with prior injection drug use, raising the question of whether the incentives would be generalizable to a population with fewer substance users, and the potential inappropriateness of the incentive schedule for patients with multiclass genotypic resistance. There was no screening for multi-class resistance prior to enrollment. Our prescription measure was “number of ART medication refills” rather than “proportion of expected refills,” so it is possible that changes may have reflected differences in prescribing intervals rather than differences in adherence.

One of the novel features of this study is that the size of the incentive is linked to future costs averted by preventing HIV infections due to greater viral load reduction. While the size of our incentive may seem large ($100 per quarter), it is important to note that this cost-savings calculation was based on extremely conservative assumptions, in particular, substantial viral load reduction from partial ART adherence prior to any intervention, risk behaviors typical of individuals who know their serostatus, and modest pre-ART viral loads. An intervention targeted at one or more high risk subgroups, in particular individuals with high pre-intervention viral loads and/or prevalent risk behaviors could pay up to $2,241 per quarter (Table 1) while still being offset by future HIV-related costs that are averted. An important limitation of our method for incentive estimation is that we did not consider costs that were unrelated to transmission (e.g., associated with development of drug resistance and/or morbidity and mortality from inadequately treated HIV) and we did not consider the incremental resource costs of the incentive program itself.

## Conclusion

We report that it is feasible to use financial incentives with the aim of reducing viral load among HIV patients in care, and to specify the incentive by requiring cost-neutrality, based on the avoided costs from downstream infections averted. Future studies are needed to assess its effectiveness, scalability, and sustainability.
